# Refractive Index Imaging Reveals That Elimination of the ATP Synthase C Subunit Does Not Prevent the Adenine Nucleotide Translocase-Dependent Mitochondrial Permeability Transition

**DOI:** 10.3390/cells12151950

**Published:** 2023-07-27

**Authors:** Maria A. Neginskaya, Sally E. Morris, Evgeny V. Pavlov

**Affiliations:** 1 Department of Molecular Pathobiology, New York University, 345 East 24th Street, New York, NY 10010, USA; sm10136@nyu.edu (S.E.M.); ep37@nyu.edu (E.V.P.); 2 Department of Medicine, Albert Einstein College of Medicine, 1300 Morris Park Ave, New York, NY 10461, USA

**Keywords:** mitochondria, mitochondrial permeability transition, mitochondrial permeability transition pore, ATP synthase, C subunit of ATP synthase, adenine nucleotide translocase, refractive index imaging, holographic imaging, cyclosporin A, bongkrekic acid

## Abstract

The mitochondrial permeability transition pore (mPTP) is a large, weakly selective pore that opens in the mitochondrial inner membrane in response to the pathological increase in matrix Ca^2+^ concentration. mPTP activation has been implicated as a key factor contributing to stress-induced necrotic and apoptotic cell death. The molecular identity of the mPTP is not completely understood. Both ATP synthase and adenine nucleotide translocase (ANT) have been described as important components of the mPTP. Using a refractive index (RI) imaging approach, we recently demonstrated that the removal of either ATP synthase or ANT eliminates the Ca^2+^-induced mPTP in experiments with intact cells. These results suggest that mPTP formation relies on the interaction between ATP synthase and ANT protein complexes. To gain further insight into this process, we used RI imaging to investigate mPTP properties in cells with a genetically eliminated C subunit of ATP synthase. These cells also lack ATP6, ATP8, 6.8PL subunits and DAPIT but, importantly, have a vestigial ATP synthase complex with assembled F1 and peripheral stalk domains. We found that these cells can still undergo mPTP activation, which can be blocked by the ANT inhibitor bongkrekic acid. These results suggest that ANT can form the pore independently from the C subunit but still requires the presence of other components of ATP synthase.

## 1. Introduction

The mitochondrial permeability transition pore (mPTP) is a large channel in the mitochondrial inner membrane [1,2,3,4,5]. In isolated mitochondria, activation of the mPTP by a high Ca^2+^ concentration leads to the free exchange of solutes of up to 1.5 kDa in size between the matrix and cytoplasm [1,6,7]. Opening of the mPTP results in a loss of mitochondrial function [8,9,10]. Both adenine nucleotide translocase (ANT) [11,12] and ATP synthase are closely involved in the formation of the mPTP [13,14]. It was previously demonstrated that the channel part of the mPTP can be formed by the C-ring of ATP synthase [15]. However, it was also established that ANT alone can form large conductance pores with properties resembling the mPTP [12,16]. Currently, it is not entirely clear which protein complex forms the mPTP pore in intact cells, and the question remains controversial [17].

We recently developed a new approach which allows observation of mPTP opening inside living cells and discriminates the mPTP from mitochondrial depolarization [18]. This approach utilizes refractive index (RI) imaging to detect the change in mitochondrial RI upon mPTP opening and the exchange of large solutes between the mitochondrial matrix and the cytoplasm. Application of RI imaging simultaneously with traditional fluorescent detection of the mitochondrial membrane potential showed that under conditions of Ca^2+^ stress, mitochondrial depolarization does not necessarily reflect mPTP opening in intact cells. Using RI imaging, we demonstrated that deletion of either ANT or ATP synthase in living cells does not prevent depolarization but leads to the elimination of the Ca^2+^-induced mPTP. These results show that, despite the presence of a channel-forming protein (either ANT or ATP synthase), its conversion into an mPTP requires the presence of a partner protein [18]. This suggests that mPTP activation at the level of intact cells might critically depend on the interactions between ANT and ATP synthase. Here, we further explore this possibility by assessing mPTP in intact cells with a genetically deleted C subunit on ATP synthase. These cells lack the ion-conducting C-ring part of ATP synthase, but contain a vestigial ATP synthase complex formed by the remaining subunits. However, the deletion of the C subunit also resulted in the absence of ATP6, ATP8, 6.8PL subunits and DAPIT in the vestigial ATP synthase complex [19].

To detect mPTP activity in intact cells, we measured changes in the mitochondrial RI using holographic tomography (referred to as RI imaging hereafter). As we described recently, solute exchange between the mitochondrial matrix and the cytoplasm due to the mPTP leads to a decrease in the RI of the mitochondria, which can be detected by the RI imaging technique [18,20,21]. We found that cells lacking the C subunit can still develop an mPTP, further supporting the notion that the presence of ATP synthase, even in its non-conducting form, is essential for mPTP activation.

## 2. Materials and Methods

### 2.1. Cell Lines

Immortalized HAP1 WT and HAP1-A12 cells with deleted a C subunit on ATP synthase were used for this study. The HAP1 cell lines were kindly provided by Professor John Walker (MRC Mitochondrial Biology Unit, University of Cambridge, Cambridge, UK). The cells were cultured as described previously [19,22]. Briefly, HAP1 cells were grown in Iscove’s Modified Dulbecco’s Medium (IMDM) supplemented with 10% heat-inactivated fetal bovine serum (HI FBS; Gibco, New York, NY, USA), 10 mL per L of antibiotic antimycotic solution (penicillin/streptomycin/amphotericin B; Sigma Aldrich, St. Louis, MO, USA) and 2 mM l-glutamine. Cells were maintained in a humidified cell incubator at 37 °C under a 5% CO_2_ atmosphere.

### 2.2. Holographic and Fluorescent Imaging

The imaging procedure followed a previously described method [18]. Cells were seeded on poly-d-lysine-coated glass coverslips and imaging was performed in Hank’s balanced salt solution (HBSS, Gibco, New York, NY, USA) at room temperature. To track changes in mitochondrial membrane potential, the fluorescent probe tetramethylrhodamine, methyl ester (TMRM, Invitrogen, Waltham, MA, USA) was used. Cells were incubated with 20 nM TMRM for 15 min in the dark at room temperature. Recordings were performed in the presence of 20 nM TMRM.

To induce mitochondrial Ca^2+^ overload and subsequent mPTP, ferutinin at a concentration of 20 μM was used. RI images (holographic reconstructions) and TMRM signals were acquired every 15 s using a 3D Cell Explorer-fluo (Nanolive, Tolochenaz, Switzerland) equipped with a 60× objective. At the end of each experiment, 10 μM of the protonophore carbonyl cyanide 4-(trifluoromethoxy) phenylhydrazone (FCCP, Sigma Aldrich, St. Louis, MO, USA) was added to induce complete depolarization of mitochondria, allowing for the determination of the minimal TMRM signal for subsequent normalization and analysis.

Cyclosporin A (CSA, Sigma Aldrich, St. Louis, MO, USA) and bongkrekic acid (BA, Sigma Aldrich, St. Louis, MO, USA), inhibitors of mPTP and ANT, respectively, were used to explore the regulation of the mitochondrial permeability transition.

### 2.3. Data Analysis

Data analysis was performed using Fiji. Multipage TIF files with Z-stack maximal intensity projections of the cells were prepared as described in [18,23]. Mitochondria were segmented using Ilastik, an interactive learning and segmentation toolkit. The area occupied by mitochondria in each cell (mitochondrial area) was estimated after segmentation. The initial mitochondrial area value in untreated cells was set to 1 for normalization of RI signals. A decrease in the RI of mitochondria was reflected by a decrease in the segmented mitochondrial area, indicating mitochondrial permeabilization.

Regions of interest (ROIs) with functional mitochondria were manually selected based on the TMRM signal. The TMRM signal was normalized as follows: the initial signal was set as 1 and the signal after the addition of FCCP and subsequent full mitochondrial depolarization was set as 0. Each data point throughout the experiment was recalculated relative to these two levels to account for changes in the TMRM signal over time. The time of half depolarization was determined as the time between the addition of ferutinin and the point where the TMRM signal dropped to 0.5.

The same ROIs were used to estimate changes in membrane potential and applied to binary segmented masks to detect changes in the mitochondrial area.

### 2.4. Statistics

Origin 2021b software (OriginLab, Northampton, MA, USA) was used for data presentation, analysis and statistics. All the data are presented as means ± standard error of the mean (SEM). The exact numbers of experiments (N) and mitochondrial areas (n, no more than two per cell) analyzed are mentioned in figure legends. A one-way ANOVA and a *t*-test were used to verify statistical significance.

## 3. Results

### 3.1. Cells Lacking C Subunit of the ATPase Can Still Undergo a Mitochondrial Permeability Transition

Previously, we demonstrated that the canonical mPTP requires the presence of both assembled ATP synthase and ANT [18]. In order to further investigate the potential interaction between ATP synthase and ANT in the context of the mitochondrial permeability transition, we employed the RI imaging approach on HAP1 cells lacking the C subunit (HAP1 C sub KO). Previous studies have shown that these cells are unable to produce the C subunit but still retain a vestigial ATP synthase complex with intact catalytic F1 and peripheral stalk domains [19]. To stimulate mPTP activation in both HAP1 WT and C sub KO cells, we utilized the ionophore ferutinin, which induces CSA-sensitive mitochondrial depolarization and permeabilization of the mitochondrial inner membrane [19,24,25,26].

**Figure 1:**
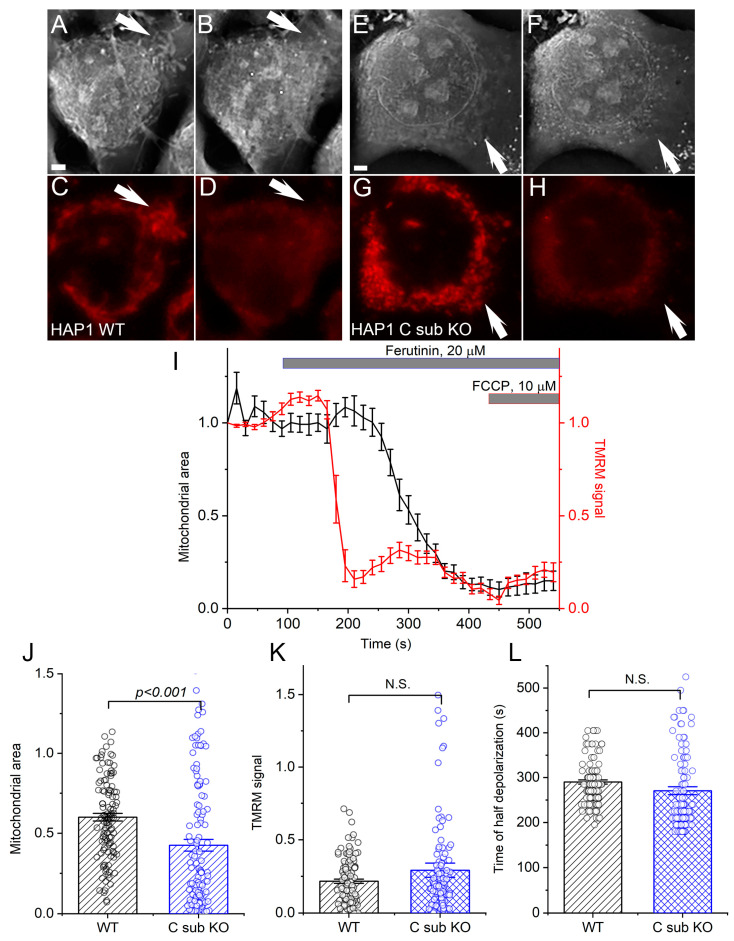
C subunit-deficient mitochondria of HAP1 cells undergo a permeability transition and depolarization when stimulated with ferutinin. (**A**–**D**) Ferutinin (20 μM) induced mitochondrial permeabilization (**A**,**B**) and depolarization (**C**,**D**) in WT HAP1 cells. (**A**,**B**) RI images before and after ferutinin addition. (**C**,**D**) TMRM fluorescence before and after ferutinin addition. Note the disappearance of mitochondria from the RI image and the decrease in the TMRM signal, indicating permeabilization and depolarization, respectively. (**E**–**H**) Ferutinin (20 μM) induced mitochondrial permeabilization (**E**,**F**) and depolarization (**G**,**H**) in HAP1 C sub KO cells. (**E**,**F**) RI images before and after ferutinin. (**G**,**H**) TMRM fluorescence before and after ferutinin. Note the disappearance of mitochondria from the RI image and the decrease in the TMRM signal, indicating mitochondrial permeabilization and depolarization, respectively. (**I**) Representative averaged traces of a single experiment showing the changes in mitochondrial area (black) and TMRM signal (red) in HAP1 C sub KO cells. Both signals were normalized to the initial level. Uncoupler FCCP was added at the end of each experiment for TMRM signal normalization. Representative of N = 11 experiments. (**J**) Mitochondrial area in RI images after the ferutinin-induced mitochondrial permeability transition. Each dot represents the mitochondrial area in the individual ROI before FCCP addition normalized to the initial level. (**K**) Normalized TMRM signal right before FCCP addition in WT and C sub KO HAP1 cells. Each dot represents the individual ROI. (**L**) Time of half depolarization of mitochondria (from ferutinin addition to 0.5 value) in WT and C sub KO HAP1 cells. N = 6, n = 113 for HAP1 WT; N = 11, n = 127 for HAP1 C sub KO. Arrows point to mitochondrial areas. Scale bars—2 μm. *T*-test.

Upon treatment with ferutinin, HAP1 C sub KO cells exhibited a loss of TMRM staining and contrast in the RI image (Figure 1E–H), similar to HAP1 WT cells (Figure 1A–D). Notably, the loss of TMRM signal and RI contrast were observed as separate events, with TMRM loss occurring before RI loss (Figure 1I). This suggests that mitochondrial depolarization precedes mPTP opening in HAP1 C sub KO cells, as was shown for HAP1 WT cells [18]. A comprehensive analysis of the area occupied by mitochondria on RI images and the TMRM signal indicated that ferutinin induced a more profound mPTP induction in HAP1 C sub KO cells compared to WT cells (Figure 1J), while the degree of depolarization was indistinguishable between the two cell types (Figure 1K–L).

These findings demonstrate that HAP1 cells can experience both mitochondrial depolarization and mPTP opening even in the absence of the C subunit of ATP synthase. Therefore, we propose that ATP synthase interacts with ANT and subsequently promotes mPTP opening through domains other than the C subunit.

### 3.2. Regulation of the Mitochondrial Permeability Transition in HAP1 WT and C Sub KO Cells

To further explore the mechanisms underlying mPTP opening, we compared the regulation of the mPTP in HAP1 WT and C sub KO cells using two known mPTP inhibitors: CSA and BA. CSA is known to inhibit mPTP formation by interacting with cyclophilin D (CypD) [6,27,28,29], a mitochondrial peptidyl-prolyl cis–trans isomerase that has binding sites on ATP synthase [30] and ANT [31]. BA is believed to inhibit the mPTP by stabilizing conformation of ANT in the m-state, where the protein is open on the mitochondrial matrix side and closed in the intermembrane space [32,33].

**Figure 2:**
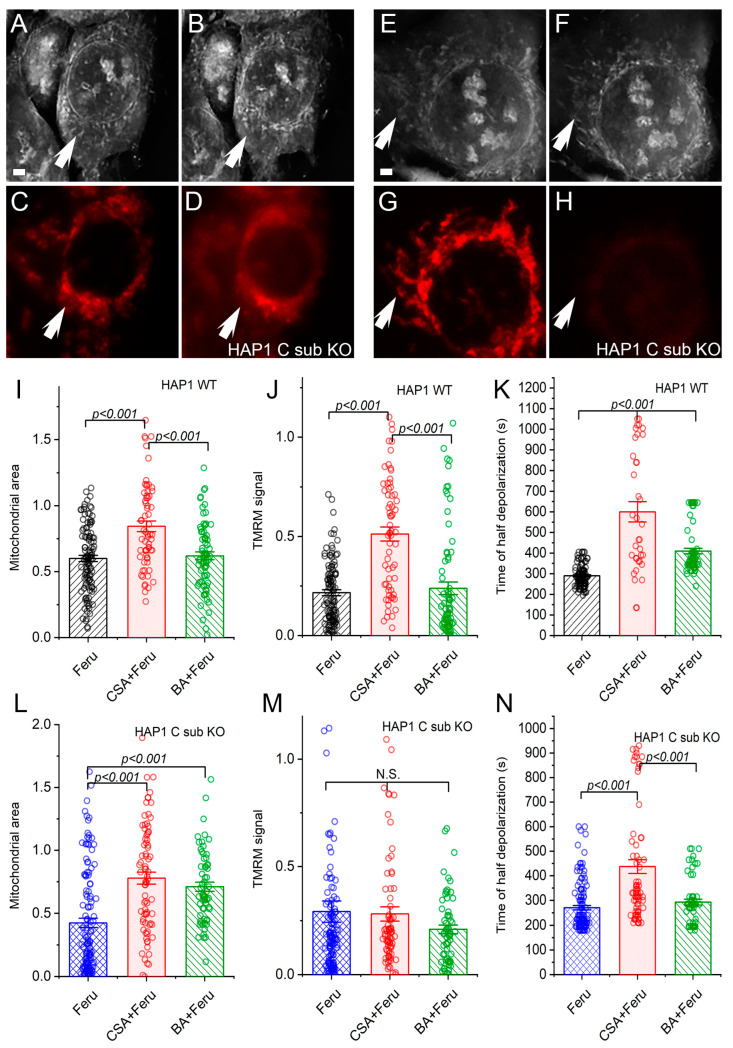
Action of inhibitors of mPTP on mitochondrial permeabilization and depolarization in WT and C sub KO HAP1 cells. (**A**–**D**) Inhibitor of mPTP CSA blocked ferutinin-induced mitochondrial permeabilization (**A**,**B**) but did not block depolarization (**C**,**D**) in HAP1 C sub KO cells. (**A**,**B**) RI images before and after ferutinin in the presence of 2 μM CSA. (**C**,**D**) TMRM fluorescence before and after ferutinin in the presence of 2 μM CSA. (**E**–**H**) Inhibitor of ANT BA blocked ferutinin-induced mitochondrial permeabilization (**E**,**F**) but did not block depolarization (**G**,**H**) in HAP1 C sub KO cells. (**E**,**F**) RI images before and after ferutinin in the presence of 2 μM CSA. (**G**,**H**) TMRM fluorescence before and after ferutinin in the presence of 2 μM CSA. Note the mitochondria in the RI image after ferutinin addition when the TMRM signal decreased, indicating unpermeabilized mitochondria with a lower membrane potential. (**I**–**K**) Mitochondrial area, remaining TMRM signal and time of half mitochondrial depolarization in HAP1 WT cells after addition of ferutinin in the presence of inhibitors of mPTP. N = 6, *n* = 113 for ferutinin; N = 3, *n* = 65 for ferutinin + CSA; N = 3, *n* = 75 for ferutinin + BA. For time of half depolarization in the ferutinin + CSA group, *n* = 36, since the TMRM signal did not decrease in the rest of the cells. (**L**–**N**) Mitochondrial area, remaining TMRM signal and time of half mitochondrial depolarization in HAP1 C sub KO cells after addition of ferutinin in the presence of inhibitors of mPTP. N = 11, *n* = 127 for ferutinin; N = 6, *n* = 79 for ferutinin + CSA; N = 4, *n* = 62 for ferutinin + BA. Each dot on the bar graphs represents the value of the normalized mitochondrial area or normalized TMRM signal in the individual ROI. Arrows point to mitochondria. Scale bars—2 μm. One-way ANOVA with Tukey post hoc test.

Both CSA and BA inhibited the loss of mitochondrial contrast in RI images, indicating the prevention of mPTP opening in HAP1 C sub KO cells (Figure 2A,B,E,F,L). In contrast, in HAP1 WT cells, only CSA completely blocked the mPTP, as evidenced by the lack of a decrease in mitochondrial area in RI images (Figure 2I), which is consistent with previous findings [18]. BA did not prevent the loss of mitochondrial contrast in RI images in WT cells (Figure 2I), suggesting the predominant role of ATP synthase in mPTP formation when stimulated with ferutinin in the presence of the C subunit.

Neither CSA nor BA could prevent mitochondrial depolarization in HAP1 C sub KO cells, as indicated by the decrease in the TMRM signal after ferutinin treatment (Figure 2C,D,G,H,M). However, CSA delayed ferutinin-induced depolarization in HAP1 C sub KO cells (Figure 2N), indicating that mitochondrial depolarization is still partially sensitive to CSA. In WT cells, CSA effectively blocked ferutinin-induced mitochondrial depolarization (Figure 2J–K), as expected. Mitochondrial depolarization was delayed but not completely blocked when HAP1 WT cells were pretreated with the ANT inhibitor BA, suggesting the possibility of a cooperative action between ATP synthase and ANT in ferutinin-induced depolarization (Figure 2K).

## 4. Discussion

The mitochondrial permeability transition, characterized by the opening of a non-selective pore in the inner mitochondrial membrane, is proposed to occur through the involvement of either ATP synthase or ANT under conditions of Ca^2+^ overload [16,22,34,35,36,37,38,39,40]. Our recent study utilizing RI imaging provided evidence that the presence of both ATP synthase and ANT in their functional forms is necessary for mPTP formation [18]. To further investigate the mechanism underlying the interaction between ATP synthase and ANT in mPTP formation, we applied the same RI imaging approach to HAP1 cells in which the C subunit of ATP synthase was genetically abolished.

We demonstrated that even in the absence of the C subunit, mitochondria within intact cells are still capable of undergoing a permeability transition and forming an mPTP. Interestingly, the mPTP in cells deficient in the C subunit is sensitive to the inhibitor of ANT (BA). These findings suggest that ANT can participate in mPTP formation independently of the C subunit of ATP synthase. However, considering that the presence of assembled ATP synthase is essential for mPTP formation [18], it is plausible to propose that ANT still relies on certain components of ATP synthase to facilitate mPTP formation. Moreover, interactions with ATP synthase could vary between isoforms of ANT, as it has been suggested that different ANT isoforms exhibit varying sensitivity to matrix Ca^2+^. This variation in sensitivity could potentially represent different populations of channels [41,42]. 

HAP1 C sub KO cells have been extensively characterized in previous studies conducted by the J. Walker lab [19]. They demonstrated that in the absence of the C subunit, these cells still exhibit mitochondrial depolarization in a manner sensitive to CSA, which is consistent with our observations in this study. Furthermore, in our investigation, in addition to mitochondrial depolarization, we observed the opening of the mPTP in HAP1 C sub KO cells, as visualized by RI imaging independently of depolarization. Importantly, the mPTP opening in these cells was also sensitive to CSA, confirming that depolarization and mPTP opening are sequential events in the process of mitochondrial permeability transitions. It is worth mentioning that DAPIT, the mitochondrially encoded subunit ATP6 and ATP8 and 6.8PL subunits were reported to be absent in HAP1 C sub KO cells [19], which excludes these subunits from potential interaction with ANT. Previous studies of mPTP in HAP1 C sub KO cells [19] and HAP1 C + δ double KO cells [24] with a fluorescent approach only challenged the participation of ATP synthase in the formation of the pore. However, this study and our previously reported investigations stress the important role of ATP synthase in mPTP formation. Further, this work highlights that ATP synthase interaction with ANT plays a critical role in the permeability transition even in the absence of the channel forming C-ring of ATP synthase.

Multiple electrophysiological studies have confirmed that both ANT and ATP synthase, when purified, can form an mPTP-like channel in artificial lipid membranes [11,12,13,14,35,40,43]. Additionally, it has been shown that the purified C-ring of ATP synthase can form a leak channel, which can be inhibited by the ATP synthase F1 complex [15]. Therefore, there is no doubt regarding the ability of both protein complexes, or even their individual components, to form a pore in artificial membranes. The possibility of ATP synthase and ANT forming a pore in intact cells emerged following patch clamp experiments on mitochondria isolated from HAP1 C sub KO cells. These experiments revealed that the absence of the C subunit resulted in the opening of a channel in response to Ca^2+^ stress. Importantly, this channel could be effectively blocked by BA [22]. Subsequently, it was suggested that either ANT or ATP synthase could form an mPTP with slightly different electrophysiological characteristics, depending on the abundance of these protein complexes in the native membrane [10,33]. 

Recently, patch clamping of proteoliposomes with reconstituted submitochondrial vesicles containing both ANT and ATP synthase demonstrated the presence of a channel with mPTP-like properties. Inhibition of ATP synthase or ANT reduced the conductance of this channel, indicating cross-inhibition between the ATP synthase and ANT pores [44]. 

The whole mitoplast patch clamp, which measures the integral current through the mitochondrial inner membrane, detected the BA-sensitive component of the current when the mitochondrial permeability transition was induced by oxidative stress but not by excessive Ca^2+,^ further evidencing the contribution of ANT towards the mPTP. On the other hand, APD, which blocks both ATP synthase and ANT channels, completely blocked the whole mitoplast mPTP current, supporting the notion that both ANT and ATP synthase likely contribute towards mPTP activity in the native membrane [43].

Our current study showed that, unlike in WT cells, mPTP opening in intact C subunit-deficient cells is sensitive to BA, suggesting that ANT plays a predominant role in mPTP formation when the pore-forming component of ATP synthase is absent. Interestingly, we observed that BA delayed ferutinin-induced mitochondrial depolarization in WT cells, indicating a regulatory role of ANT even in the presence of the C subunit and further supporting an interaction model of the mPTP and the cooperativity of these two channels. 

## 5. Conclusions

**Figure 3:**
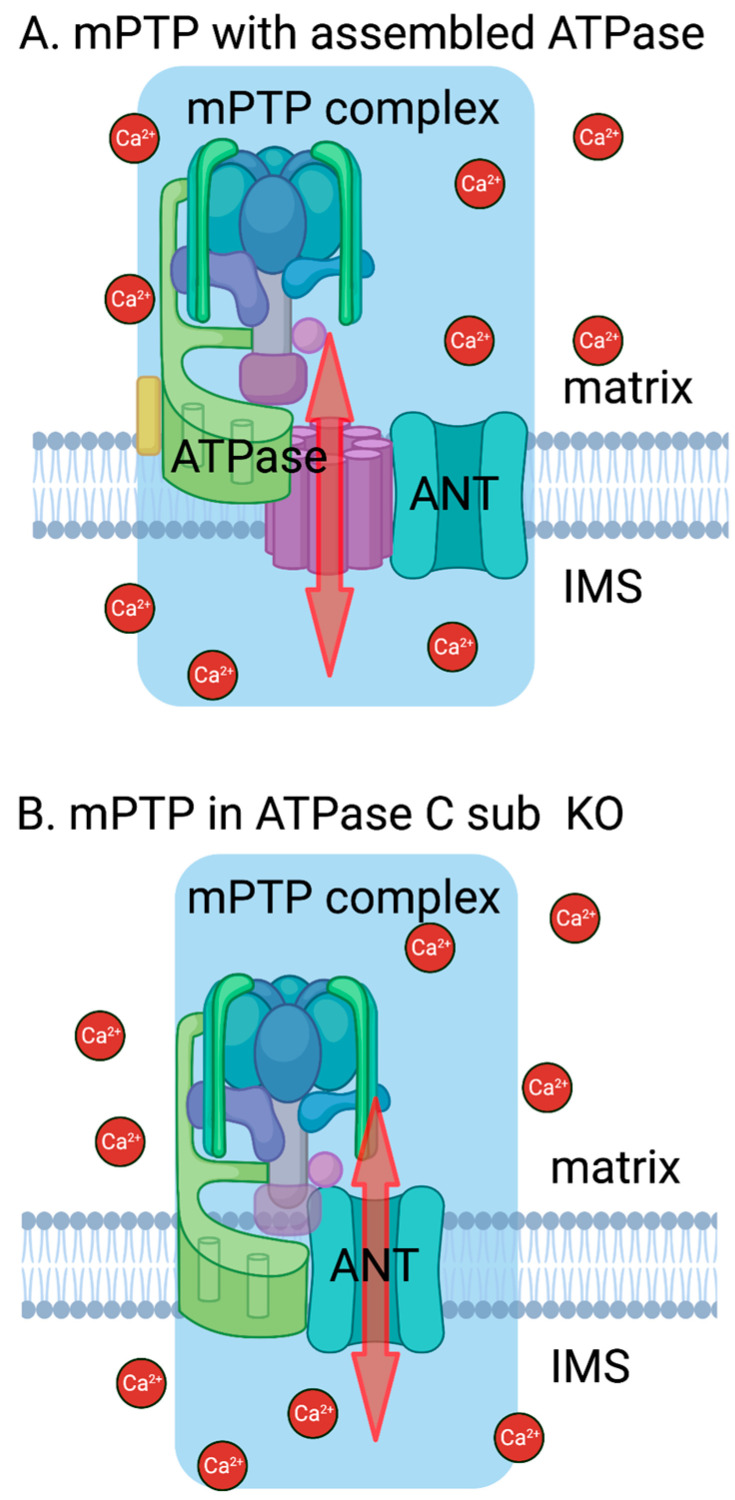
Model of mPTP formation. In conditions of mitochondrial Ca^2+^ overload, an mPTP is formed by a complex involving ATP synthase and ANT interacting with each other. (**A**) When both protein complexes (ATP synthase and ANT) are fully assembled and functional, ATP synthase takes on the role of the permeable part of the pore (adapted from [45,46]). (**B**) In the absence of the ion-conducting part of ATP synthase in the membrane, ANT acts as the permeable component of the mPTP complex. ATPase refers to ATP synthase and IMS refers to the intermembrane space. Created with BioRender.com.

In summary, we found that cells lacking an ATP synthase C subunit can still undergo Ca^2+^-induced mPTP formation. In C sub KO cells, ANT is likely responsible for pore opening, since mPTP was blocked by both CSA and BA (Figure 3). Overall, these results suggest that the interaction between ATP synthase and ANT plays an important role in formation and regulation of the mPTP.

## 6. Limitation of the Study

We did not observe inhibition of mitochondrial depolarization by BA in the C sub KO cells, which has been previously reported. This discrepancy could potentially be attributed to the higher concentration of ferutinin used in the current study, which might have masked the inhibition of mitochondrial depolarization. 

We cannot completely rule out mitophagy as a subsequent event following mitochondrial depolarization, as depolarization has been known to trigger mitophagy [47]. This intriguing direction presents a potential avenue for future studies, which we are planning to pursue. 

However, we believe that the loss of matrix content observed in the current study is indicative of a mitochondrial permeability transition for several reasons. Firstly, it is CSA sensitive, suggesting the involvement of the mitochondrial permeability transition pore. Secondly, the time interval between mitochondrial depolarization and permeabilization was less than 100 s, which may not be sufficient for the formation of a mitophagosome based on previously reported times of 6–10 min in the case of starvation-induced mitophagy [48] and up to 40 min in stress-induced mitophagy [49,50]. Furthermore, mitochondrial fission, which typically precedes mitophagosome formation, was not observed in the current study.

In the future, it will be important to further explore the limitations and advantages of the RI imaging approach, which we believe offers a number of unique advantages, in comparison to other assays for detecting the mPTP.

## Data Availability

All the data supporting the study is presented in the current manuscript. The raw data, along with analysis protocols, are available from the corresponding authors upon request.
